# Supplementary material for: A neural circuit for context-dependent multimodal signaling in Drosophila
Source: Nat Commun. 2025 Oct 27;16:9472. doi: 10.1038/s41467-025-64907-9 (PMC12559195; doi:10.1038/s41467-025-64907-9)
Supplement: Supplementary file 1 — Supplementary Information [file 41467_2025_64907_MOESM1_ESM.pdf]

Supplementary information

Mathematical details of circuit model

pC2l

The population activity of the pC2l neurons is a copy of their optogenetic input: $r_{pC2l} = I_{opto \rightarrow pC2l}$. Optogenetic input was modeled as rectangular pulses with the same duration as used in the experiments (5 s, interleaved by a pause of 120 s). We assumed a logarithmic mapping from LED intensity to input current (14, 27, 42, 83 mW/cm^2 -> 0.5, 0.6, 1.1, 1.4 nA).

P1a

The inputs to P1a are given by:

$$I_{P1a} = I_{opto \rightarrow P1a} + \Theta(r_{pC2l} - \theta_{pC2l \rightarrow P1a}) w_{pC2l \rightarrow P1a} \tag{1}$$

where $I_{opto \rightarrow P1a}$ is the input from optogenetic activation (or sensory cues), and r_{pC2l} is the input from pC2l which is passed through a threshold-linear function

$$\Theta(x) = \begin{cases} 0 & x \leq 0 \\ x & x > 0 \end{cases}$$

after subtraction of a threshold term $\theta_{pC2l \rightarrow P1a}$. The threshold ensures that weak activation of pC2l is insufficient to drive offset vibrations via P1a (Fig. S5F). As for pC2l, we assumed a logarithmic mapping from LED intensity to input current (14, 27, 42, 83 mW/cm^2 -> 0.12, 0.16, 0.20, 0.24 nA). The response of P1a is given by

$$\frac{dr_{P1a}}{dt} = (-r_{P1a} + I_{P1a} w_{I_{P1a}} + s_{P1a} w_{s_{P1a}}) / \tau_{r_{P1a}} \tag{2}$$

$$\frac{ds_{P1a}}{dt} = (-s_{P1a} + r_{P1a}) / \tau_{s_{P1a}} \tag{3}$$

where r_{P1a} is a continuous variable proportional to the population firing rate of the P1a neurons, I_{P1a} are the external inputs to P1a (Eq. 1) with weight $w_{I_{P1a}}$, s_{P1a} is the input from a slow variable with weight $w_{s_{P1a}}$, and $\tau_{r_{P1a}}$ is the membrane time constant. The slow decay of P1a activity [1] is replicated by a positive feedback loop between r_{P1a} and a slow variable, s_{P1a}. The slow variable could represent cell-intrinsic mechanisms arising from slow calcium dynamics coupled with calcium-activate sodium channels. The slow variable receives input from P1a, r_{P1a}, and is integrated with time constant $\tau_{s_{P1a}}$. Before being passed on to downstream partners, the output of P1a is transformed using a static logarithmic nonlinearity to mimic response saturation $r_{P1a} = \log(1 + 2 r_{P1a})$.

Neuron	Component	Parameter name	Parameter value
P1a	Response r_{P1a}	Threshold for input from pC2l $\theta_{pC2l \rightarrow P1a}$	7 nA
		Weight for input from pC2l $w_{pC2l \rightarrow P1}$	0.15
		Weight for input to P1a $w_{I_{P1a}}$	0.8
		Weight for slow variable $w_{s_{P1a}}$	0.8
		Time constant $\tau_{r_{P1a}}$	0.7 s
	Slow variable s_{P1a}	Time constant $\tau_{s_{P1a}}$	0.1 s

Table 1: Model parameters for P1a.

Recurrent neural network

While the slow variable, s_{P1a} (Eq. 3), reproduces the known slow decay of P1a activity [1], a recurrent neural network (RNN) downstream of P1a generates persistent signaling over tens of

seconds after P1a activation [2]:

$$\frac{dI_{RNN}}{dt} = (-I_{RNN} + \Theta(r_{P1a} - \theta_{P1a \to RNN})/\tau_{I_{RNN}} \tag{4}$$

$$\frac{dr_{RNN}}{dt} = (-r_{RNN} + I_{RNN} + p_{RNN} w_{p_{RNN} \to r_{RNN}})/\tau_{r_{RNN}} \tag{5}$$

$$\frac{dp_{RNN}}{dt} = (-p_{RNN} + r_{RNN})/\tau_{p_{RNN}} \tag{6}$$

External input to the RNN, I_{RNN}, from P1a is passed through a threshold-linear function with threshold $\theta_{P1a \to RNN}$ and integrated with time constant $\tau_{I_{RNN}}$. The threshold ensures that only strong activation of P1a elicits persistence, not the weak activation from pC2l. Input from the recurrent pool, p_{RNN}, is integrated with weight $w_{p_{RNN} \to r_{RNN}}$ and together with external input, I_{RNN}, integrated with a time constant $\tau_{r_{RNN}}$. The recurrent pool receives input from the RNN itself and has a time constant $\tau_{p_{RNN}}$.

Neuron	Component	Parameter name	Parameter value
RNN	Inputs I_{RNN}	Threshold for input from P1a $\theta_{P1a \to RNN}$	1.6 nA
		Time constant $\tau_{I_{RNN}}$	16 s
	Response r_{RNN}	Weight for recurrence $w_{p_{RNN} \to r_{RNN}}$	0.96
		Time constant $\tau_{r_{RNN}}$	0.7 s
	Recurrence p_{RNN}	Time constant $\tau_{p_{RNN}}$	2 s

Table 2: Model parameters for the recurrent neural network (RNN).

Descending neurons pIP10 and DNvib

The pIP10 neuron integrates input from the RNN and from pC2l, mutual inhibition from DNvib, adaptation, and noise:

$$\frac{dr_{pIP10}}{dt} = -(r_{pIP10} + r_{RNN} w_{RNN \to pIP10} + r_{pC2} - a_{pIP10} - m_{DNvib} w_{m_{DNvib}} + \eta_{pIP10})/\tau_r \tag{7}$$

where r_{pIP10} is the activity of pIP10, r_{RNN} is the input from the RNN with weight $w_{RNN \to pIP10}$, r_{pC2} is the input from pC2l, a_{pIP10} is an inhibitory adaptation current (see eq. 9 below), m_{DNvib} is an inhibitory input from DNvib with weight $w_{m_{DNvib}}$, η_{pIP10} is Gaussian noise (see eq. 10 below), and τ_r is an integration time constant.

Similar to pIP10, DNvib integrates inputs from the RNN and P1a, mutual inhibition from pIP10, adaptation and noise:

$$\frac{dr_{DNvib}}{dt} = -(r_{DNvib} + r_{RNN} + r_{P1a} - a_{DNvib} - m_{pIP10} w_{m_{pIP10}} + \eta_{DNvib})/\tau_r \tag{8}$$

where r_{DNvib} is the activity of DNvib, r_{RNN} is the input from the RNN, r_{P1a} is the input from P1a, a_{DNvib} is an inhibitory adaptation current, m_{pIP10} is an inhibitory input from pIP10 with weight $w_{m_{pIP10}}$, η_{DNvib} is Gaussian noise (see eq. 10 below), and τ_r is an integration time constant.

To enable bistable dynamics with noise-induced switching between song and vibration after activation of P1a, we added an adaptation current and noise to pIP10 (eq. 7) and DNvib (eq. 8) [3]. The adaptation is modeled as negative feedback:

$$\frac{da_i}{dt} = -(a_i + r_i)/\tau_a \tag{9}$$

where a_i is the adaption current for neuron i, r_i is activity of neuron i, and the adaptation time constant is τ_a. Gaussian noise η with time constant τ_η and standard deviation σ_η was given by:

$$\frac{d\eta}{dt} = -\eta/\tau_\eta + \sigma_\eta * \sqrt{2/\tau_\eta} * N(0,1) \tag{10}$$

$N(0,1)$ is a random variable with zero mean and unit variance.

During integration, r_{pIP10} and r_{DNvib} are passed through a nonlinearity Σ which limits their activity to an upper bounds of ω:

$$\Sigma = \begin{cases} x & x \leq \omega \\ \omega & x > \omega \end{cases}$$

Mutual inhibition downstream of pIP10 and DNvib

Mutual inhibition downstream of pIP10 and DNvib is based on a canonical model of bistable perception [3]. In this model, switching arises from adaptation (eq. 9) and noise (eq. 10) in the response of pIP10 and DNvib. We implemented the mutual inhibition via inhibitory interneurons m_{DNvib} and m_{pIP10}, respectively. Only m_{pIP10} adapts to speed up the dynamics of the inhibitory inputs from DNvib to pIP10 which are otherwise too slow to mediate strong and fast inhibition of song from DNvib:

$$\frac{dm_{pIP10}}{dt} = (-m_{pIP10} + r_{DNvib} w_r - a_{m_{pIP10}} w_{a_{m_{pIP10}}})/\tau_m \tag{11}$$

$$\frac{dm_{DNvib}}{dt} = (-m_{DNvib} + r_{pIP10} w_r)/\tau_m \tag{12}$$

Both m_{pIP10} and m_{DNvib} integrate their external inputs with weight w_r, and have a time constant τ_m. For m_{pIP10}, $a_{m_{pIP10}}$ is the adaptation current with weight $w_{a_{m_{pIP10}}}$ and an adaptation time constant $\tau_{a_{m_{pIP10}}}$ (eq. 9).

Neuron	Component	Parameter name	Parameter value
pIP10	Response r_{pIP10}	Weight for input from RNN $w_{RNN\to pIP10}$	1.6
		Weight for mutual inhibition from DNvib $w_{m_{DNvib}}$	10
	Nonlinearity Σ_{pIP10}	Saturation ω_{pIP10}	20
DNvib	Response r_{DNvib}	Weight for input from P1a $w_{P1a\to DNvib}$	1.5
		Weight for input from RNN $w_{RNN\to DNvib}$	1.92
		Weight for mutual inhibition from pIP10 $w_{m_{pIP10}}$	1.5
	Nonlinearity Σ_{DNvib}	Saturation ω_{DNvib}	3
pIP10 or DNvib	Response r_{pIP10} or r_{DNvib}	Time constant τ_r	1 s
	Adaptation a_{pIP10} or a_{DNvib}	Time constant τ_a	5 s
Mutual inhibition from DNvib or pIP10	Response m_{pIP10} or m_{DNvib}	Weight for input from DNvib or pIP10 w_r	0.001
		Time constant τ_m	1 s
	Adaptation $a_{m_{pIP10}}$	Time constant of adaptation $\tau_{a_{m_{pIP10}}}$<br>Weight for input from adaptation $w_{a_{m_{pIP10}}}$	1 s<br>10000

Table 3: Model parameters for pIP10 and DNvib.

Model fitting and simulation

The differential equations were solved numerically with the Euler method and a time step of 1 ms, accelerated using just-in-time compilation with `numba`. The model was fitted by manually adjusting the parameters.

Model manipulations

For ablating recurrence (Fig. S10D–F), we set the weights for inputs from the RNN in pIP10 and DNvib, $w_{RNN\to pIP10}$ and $w_{RNN\to DNvib}$ to zero. For ablating mutual inhibition (Fig. S10G–I) we set the weights for inputs from the mutual inhibition, $w_{m_{DNvib}}$ and $w_{m_{pIP10}}$ to zero. Effects of sexual satiation in the model (Fig. S10J–L) were reproduced by changing 1) the gain of inputs to pC2l from 1.0 to 0.6, 2) the weight for the slow variable in P1a, $w_{s_{P1a}}$, from 0.8 to 0.75, and 3) the weight for recurrent inputs to RNN, $w_{p_{RNN}\to r_{RNN}}$, from 0.96 to 0.75.

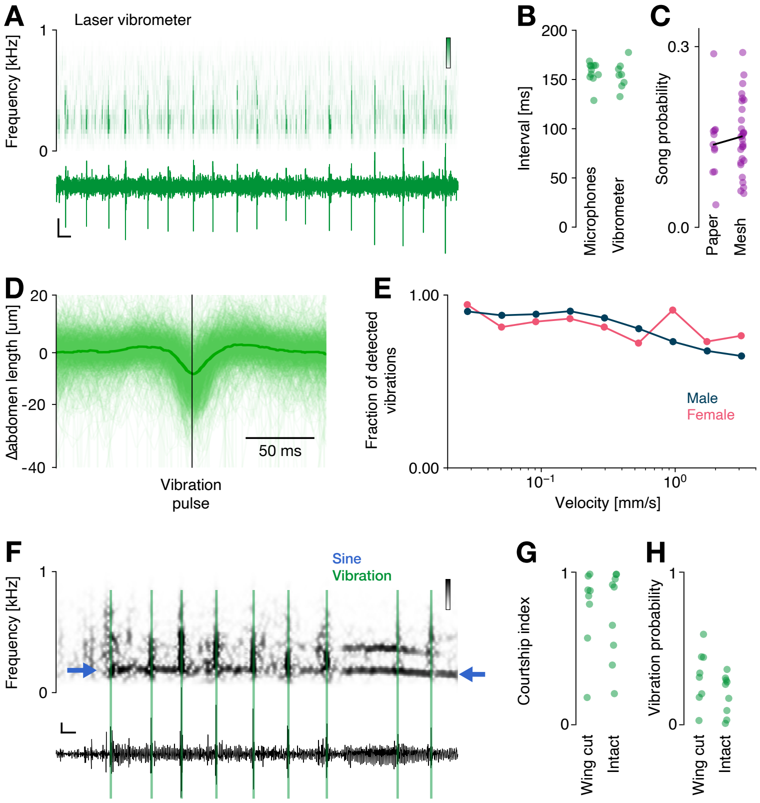

Figure S1: Vibrations can be reliably recorded using a microphone array.
A Vibrations recorded using a laser vibrometer (bottom) and the corresponding spectrogram (top). Vertical and horizontal scale bar corresponds to 20 nm/s and 100 ms.
B Intervals between vibrations recorded using laser vibrometry (155±13 ms, N=8 flies) and microphones (160±11 ms, N=11 flies) are similar (p=0.40, two-sided Mann-Whitney U test). Dots correspond to the median vibration intervals of individual males. Intervals >360 ms were excluded.
C Probability of song during courtship recorded in the same 16-microphone chamber with paper (13.7±0.5% (median±IQR), N=11 pairs) and mesh (15.1±0.9%, N=29 pairs) substrates (p=0.61, two-sided Mann-Whitney test).
D Length of the abdomen (distance between thorax center and abdomen tip) extracted from SLEAP tracked male poses aligned to vibration pulses detected on the microphones. Individual green lines show individual vibrations, the thick green line is the average over N=747 vibrations.
E Probability of detecting vibration within 0.1 s of male quivering as a function of male (blue) and female (pink) velocity. We binned velocities into 9 logarithmically spaced bins between 0.2 and 2 mm/s and calculated the fraction of detected vibrations. Over all bins, detection probability is at or above 0.80. Thus, the recording system enables reliable recoding of vibrations in stationary and walking flies.
F Microphone trace (bottom) and spectrogram (top) showing a rare overlap between sine song (dark vertical bands in the spectrogram) and vibrations (green). Vertical and horizontal scale bar corresponds to 0.1 V and 50 ms.
G Wing cut males court as much as intact males (courtship index wing cut 0.86±0.25 and intact 0.90±0.27, p=0.78, two-sided t-test).
H Wing cut males vibrate as much as intact males. Probability of vibration during courtship in wing cut and intact males: 0.32±0.16 and 0.26±0.12 (p=0.14, two-sided t-test, N=8 wing-cut, N=9 intact males).

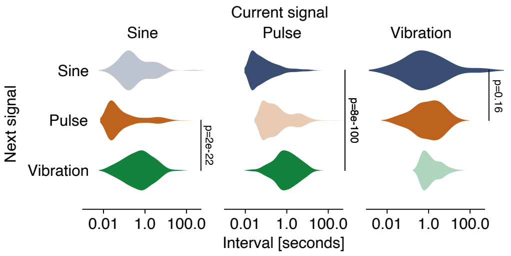

Figure S2: Song and vibrations are temporally separated.
Pauses between sine songs, pulse trains, and vibration trains. The song modes are interleaved by much shorter pauses than song and vibration. This is consistent with song and vibration being produced in distinct behavioral contexts (sine to pulse 0.04±0.16 s (median±IQR), pulse to sine 0.06±0.14 s, sine to vibration 0.57±1.16s, pulse to vibration 0.94±1.94 s).

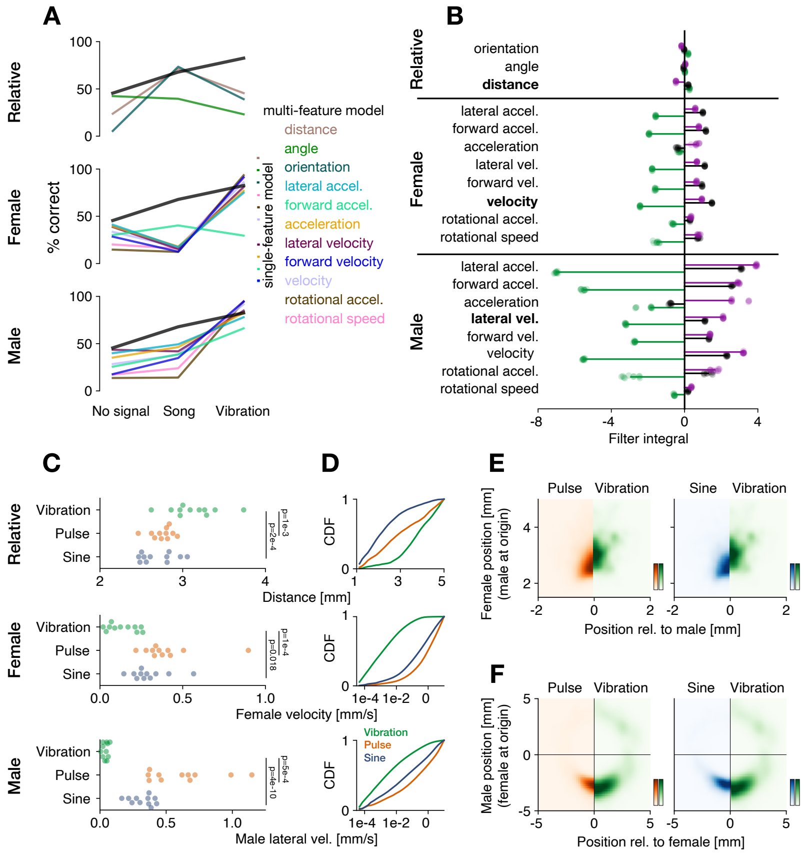

Figure S3: Males vibrate when slow and sing when close to females and moving.
A Predictive performance (% correct) of multi-feature model (black, Fig. 2C) and single-feature models for predicting no signal, song, and vibration. Same data as Fig. 2F, but color-coded by feature (see legend).
B Integral of linear filters for models fitted with single cues (same models as Fig. 2D–H). Male and female speed-related cues tend to have filters with negative integrals for vibration (green) and positive integrals for song (purple) and no signal (black). Dots correspond to filter integral from 10 fits of models with independent train-test splits, horizontal lines connect x=0 to mean over the 10 fits. Same data as Fig. 2G but for all features.
C Most predictive relative (male-female distance, top), female (velocity, middle), and male (lateral velocity, bottom) cues during sine (blue), pulse (orange), and vibration (green). Individual dots show the average value for each of 11 pairs. Distance for sine (2.6±0.3 mm, median±IQR), pulse (2.8±0.1 mm), and vibration (3.1±0.3 mm). Male lateral velocity when producing sine (0.36±0.12 mm/s), pulse (0.62±0.29 mm/s), and vibration (0.05±0.03 mm/s). Female velocity when producing sine (0.27±0.09 mm/s), pulse (0.36±0.09 mm/s), and vibration (0.12±0.15 mm/s). P-values from Dunnet post-hoc tests of a Kruskal-Wallis test (both two-sided).
D Cumulative density functions of distance (top), male velocity (middle), and male lateral velocity (bottom) for sine (blue), pulse (orange), and vibration (green) (515076 data points of courtship pooled across N=11 pairs). Same data as Fig. 2I but with song split into pulse and sine.
E, F Position of female relative to male (E) and of male relative to female (F) for pulse (orange) sine (blue) and vibration (green). Kernel density estimate plots based on positions over whole sine songs or pulse and vibration trains (N=27160/39389/13805 data points for sine/pulse/vibration over N=11 pairs).

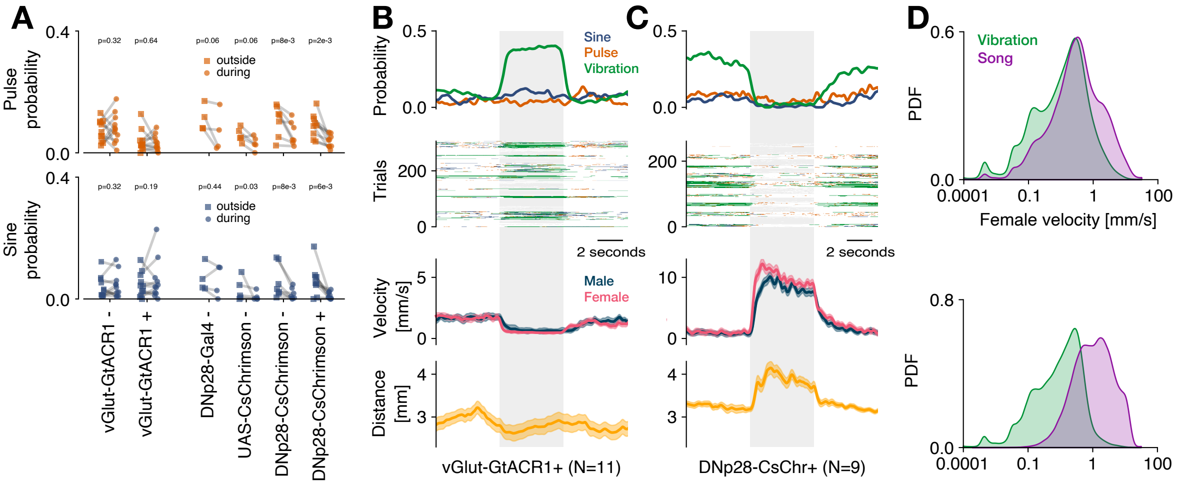

Figure S4: Manipulating female locomotion only weakly affects male singing.
A Effect of female stopping (inactivation of all motor neurons with vGlut-GtACR1) and female acceleration (activation of DNp28 neurons with CsChrimson) on pulse song (top, orange), and sine song (bottom, blue). Same data as Fig. 3B, D but with sine and pulse song. Statistics compare the signal probabilities outside (squares) and during (circles) of optogenetic stimulation for each genotype. "+" and "-" after each genotype name indicate whether flies were fed all-trans retinal, a co-factor necessary for light sensitivity in Chrimson and GtACR1 that is present only in small amounts in regular food. P-values for vGlut-GtACR1 (+ and -) from a Wilcoxon test of the hypothesis that optogenetic stimulation increases signaling. P-values for the remaining genotypes from a Wilcoxon test of the hypothesis that optogenetic stimulation decreases signaling.
B, C Trial-averaged probability of observing sine (blue), pulse (orange) and vibration (green) (top), single trial signaling (upper middle), male (blue) and female (pink) velocity (lower middle, line - mean, shaded area - standard error), and male-female distance (bottom, mean±standard error of the mean) during optogenetic inactivation of vGlut (B) and optogenetic activation of DNp28 (C). The time of optogenetic stimulation is marked as a grey shaded area. Inducing female stopping through vGlut inactivation drives vibration, but has no effect on distance and song (B). Inducing female acceleration suppresses vibrations and pulse and sine and increases the male-female distance.
D Distributions of female (top) and male (bottom) velocity during song (purple) and vibration (green). Female velocities overlap more than male velocities, indicating that male movement determines the choice between song and vibration more than female movement.

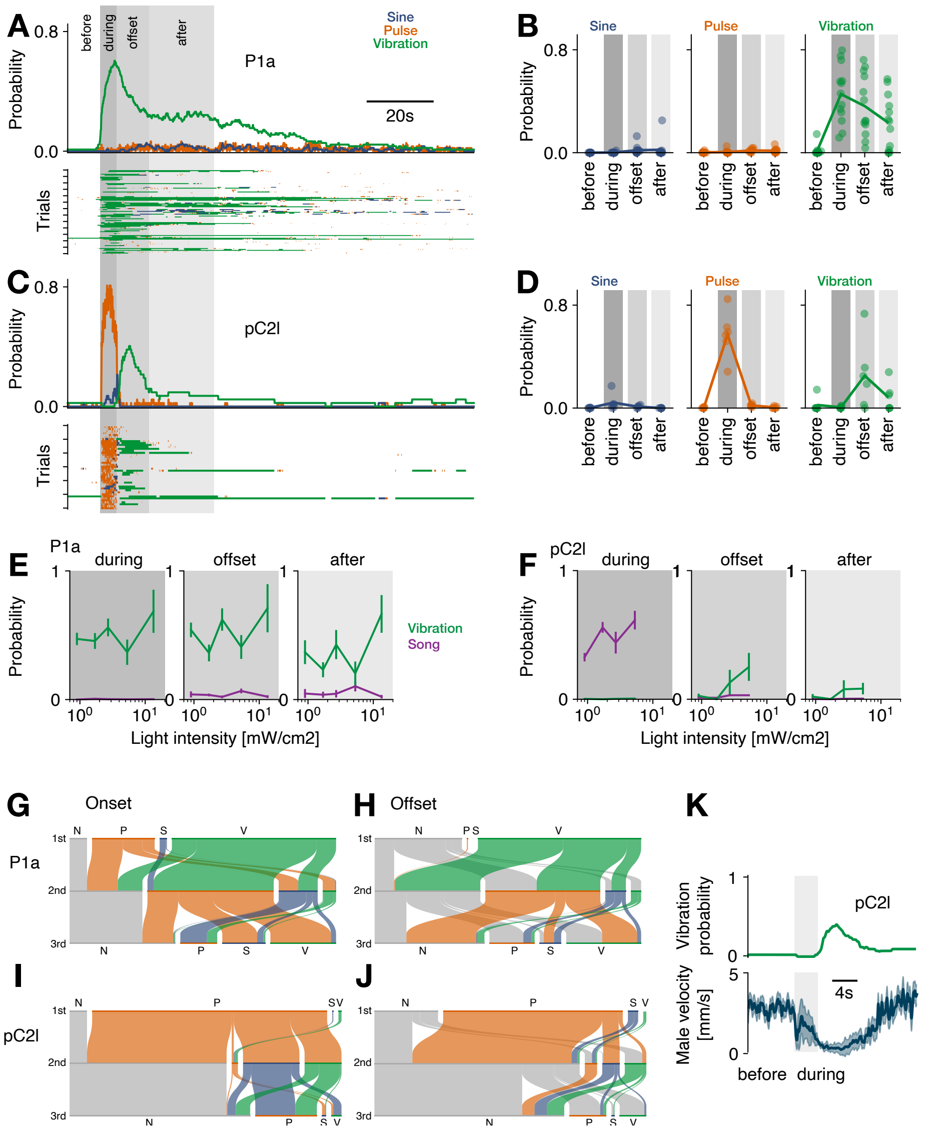

Figure S5: Activation of P1a and pC2l drives song and vibration.

A Trial average probability (top) and single trial raster (bottom) for sine (blue), pulse (orange), and vibration (green) in response to optogenetic activation of P1a in solitary males (27 mW/cm^2, N=13 flies, 7 trials/fly). Gray shaded areas delimit the epochs analysed in D. Same as Fig. 4C but song is split into sine and pulse modes.

B Probability of observing sine (left), pulse (middle), and vibration (right) in different epochs surrounding P1a activation.

C Same as A but for optogenetic activation of pC2l in solitary males (83 mW/cm^2, N=6 flies, 7 trials/fly).

D Same as B but for pC2l activation.

E, F Probability of observing song (purple) and vibration (green) in different epochs surrounding the activation of P1a (E) or pC2l (F) at different intensities (625 nm).

G–J Signal sequences produced after the onset (G, J) and offset (H, J) of optogenetic activation of P1a (G, H) and pC2l (I, J). The Sankey diagrams show the transitions between the first three signal types (N - no signal, grey; P - pulse song, orange; S - sine song, blue; V - vibration, green). The width of connecting bands is proportional to the transition probability between pairs of signals. Same data as A, C.

K Vibration probability (green) and male velocity (blue, mean±standard deviation over N=13 males with 7 trials each) in response to optogenetic activation of pC2l. Same data as C.

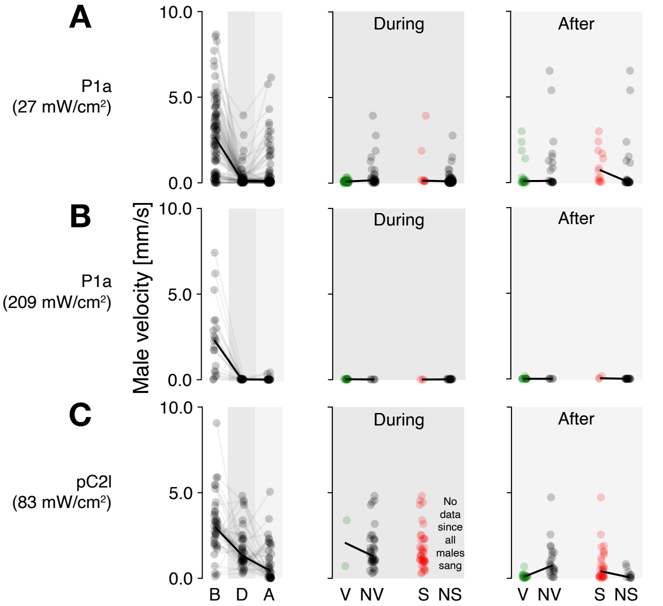

Figure S6: Effect of neuronal activation on locomotion and signaling.
A Left: Male velocity before (B), during (D), and after (A) optogenetic activation of P1a. Dots correspond to trials, lines connect the medians for each epoch. Middle/right: Velocity of males that vibrating (green, V), non-vibrating males (black, nV), singing (red, S), and non-singing (NS, black) males during (middle) and after (right) activation (27 mW/cm^2, N=13 flies, 7 trials/fly).
B Same as A but for stronger optogenetic activation of P1a (209 mW/cm^2, N=3 flies, 7 trials/fly).
C Same as A but for optogenetic activation of pC2l in solitary males (83 mW/cm^2, N=6 flies, 7 trials/fly).

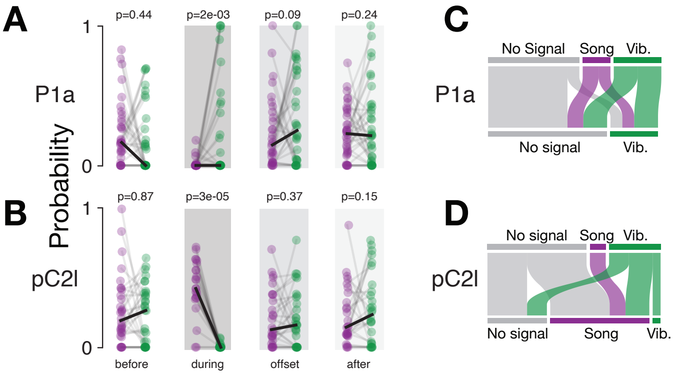

Figure S7: Activation of P1a and pC2l in males courting a female.
A, B Comparison of the probability of song (purple) and vibration (green) upon activation of P1a (A) and pC2l (B) in a male courting a female. Same data as in Fig. 5C, D. P-values from Wilcoxon tests (before: two-sided; during: P1a more vibration than song, pC2l less vibration; offset and after: more vibration than song; all hypotheses based on the results of activation in solitary males in Fig. 4 C–F).
C, D Transitions between song, vibration and silence when P1a (C) or pC2l (D) are activated optogenetically in males courting a female. After P1a activation, all males either vibrate or stop signaling. After pC2l activation, vibrating males tend to start singing or stop signaling.

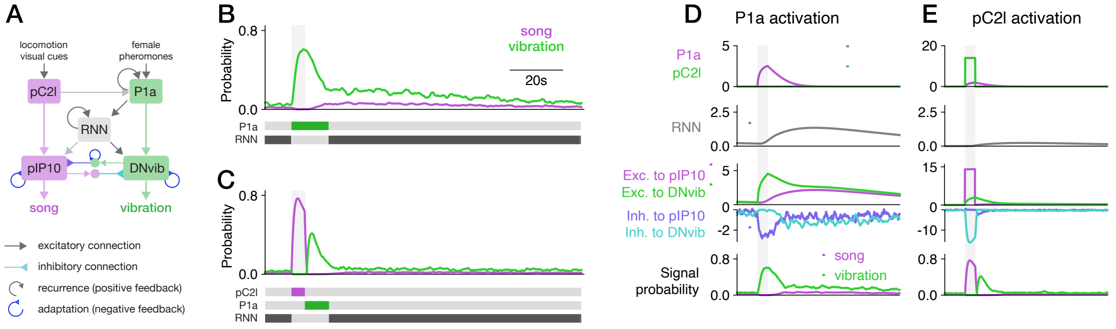

Figure S8: Detailed diagram of the network model.
A Detailed network diagram for the model. Gray and blue arrows with straight and inverted heads indicate excitation and inhibition, respectively. Circular arrows indicate positive feedback (recurrence) and negative feedback (adaptation). Colors denote the signal driven during activation of each neuron (purple - song, green - vibration).
B, C Schematic diagram of which neurons drive which signals in different phases during activation of P1a (B) and pC2l (C). P1a drives vibrations during and at the offset of P1a activation. pC2l drives song during activation. P1a drives vibration at the offset of pC2l activation. The recurrent neural network drives signaling in the persistent phase, starting 10 seconds after activation.
D Activity of individual neurons in the model during activation of P1a. Optogenetic activation of P1a decays slowly because of intrinsic processes (purple, top) and induces persistent activity in the RNN (grey, 2nd row). DNvib is directly activated by P1a (green, 3rd row), which drives strong vibrations during and immediately after P1a activation (green bottom). The RNN kicks in later to provide persistent inputs to DNvib and to pIP10 (3rd row). Strong activation of the DNvib during P1a activation drives strong inhibition to pIP10 (violet, 4th row) and thereby suppresses song during P1a activation. Inhibition from pIP10 to DNvib only kicks in later (cyan, 4th row) and enables noise-induced switching between song and vibration during the persistent phase.
E Optogenetic activation of pC2l drives pC2l activity but also weakly activates P1a (purple and green, top). The P1a activity is too weak to strongly activate the RNN (grey, 2nd row), thereby preventing persistent signaling. During pC2l activation, pIP10 is strongly activated by pC2l and drives singing (purple, 3rd row). At the same time pIP10 strongly inhibits DNvib (cyan, 4th row) which suppresses vibrations. DNvib gets input from the slower P1a activity, which outlasts the pC2l activity and the inhibition from pIP10 (green, 3rd row). The slowly decaying P1a activity then drives at the offset of pC2l activation (bottom).

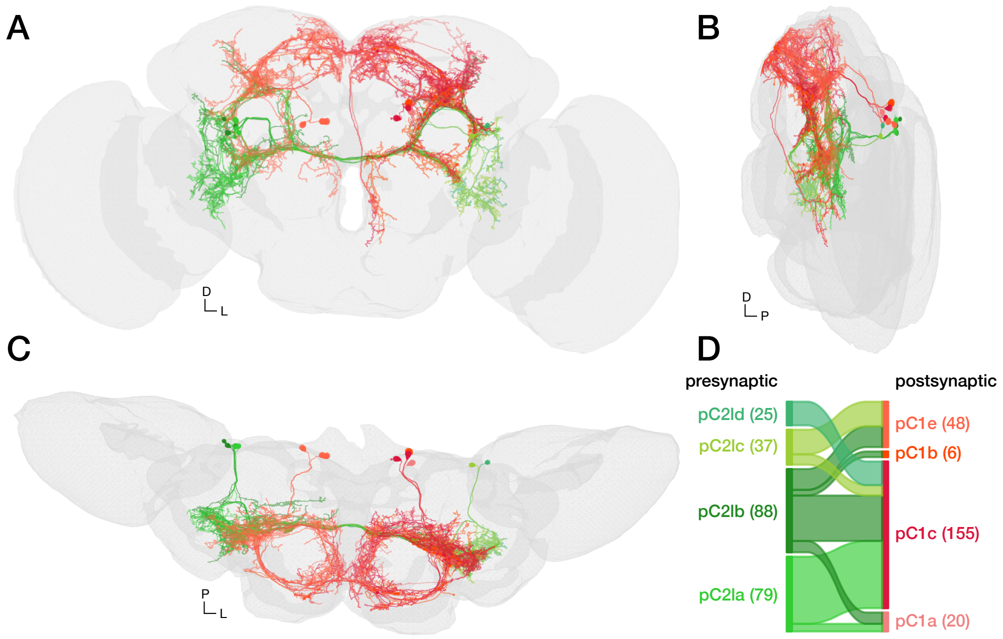

Figure S9: Connections between pC2l and pC1 in the flywire connectome.
A–C Frontal (A), lateral (B), and dorsal (C) view of pC2l (green shades) connected to pC1 neurons (red shades) in the connectome of the female brain. The P1a neurons are a male-specific subtype of the pC1 neurons in the female. Different shades of green and red indicate different subtypes of pC2l (a–d) and pC1 (a–e), respectively (color code in D). Grey shows a volume rendering of the fly brain.
D Connectivity between different subtypes of pC2l (presynaptic) and pC1 (postsynaptic) neurons. Line width is proportional to synapse count for each type of connection. Numbers beside each subtype indicate the number of outgoing (left) and incoming (right) synapses. In the female brain, there are in total 229 cholinergic synapses between 4 pC2l and 4 pC1 subtypes. It is thus likely that similar connections exist between pC2l and P1a in the male.

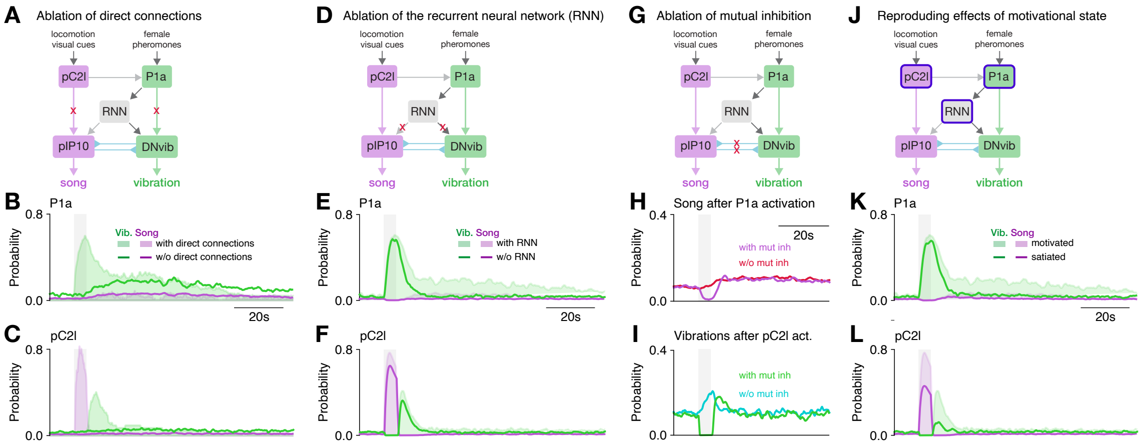

Figure S10: Ablation experiments and impact of motivation state in the circuit model.
A Testing the role of direct connections between P1a and DNvib and between pC2l and pIP10 in the model through ablation (red crosses mark ablated connections).
B, C Song (purple) and vibration (green) for activation of P1a (B) and pC2l (C) in an intact model (shaded areas) and in a model without direct connections to pIP10 and DNvib (lines) (compare data in Fig. 4C, E). Removing direct connections removes vibrations evoked during and shortly after activation of P1a (B) as well as song and vibration produced during and after pC2l activation (B). Sustained song and vibration are not affected.
D Testing the role of the recurrent neural network (RNN) in the model by removing the connections from the RNN to pIP10 and DNvib (red crosses).
E, F Song (purple) and vibration (green) for activation of P1a (E) and pC2l (F) in an intact model (shaded areas) and in a model without an RNN (lines) (compare data in Fig. 4C, E). Ablating the RNN predominantly reduces the persistent signaling after activation in P1a.
G Testing the role of mutual inhibition in the network model by removing the inhibitory connections between pIP10 and DNvib (red crosses).
H, I Song upon P1a activation (H) and vibrations upon pC2l activation (I) in an intact network (purple and green lines) and in a network without mutual inhibition (red and cyan lines) (compare data in Fig. 5C–D). Without mutual inhibition signals (song/vibration) are not suppressed during activation of P1a/pC2l.
J Modeling the impact of sexual satiation on the circuit. Sexual satiation was modeled by reducing the excitability in pC2l, the slow decay P1a as well as the recurrent excitation in the RNN.
K, L Song (purple) and vibration (green) for activation of P1a (K) and pC2l (L) in naive, sexually motivated males (shaded areas) and in sexually satiated males (lines). In the model responses of pC2l to activation are reduced, as are the persistent vibrations after activation of P1a and pC2l. This is consistent with the experimental data in Fig. 5I–J.
